# HyTexiLa: High Resolution Visible and Near Infrared Hyperspectral Texture Images

**DOI:** 10.3390/s18072045

**Published:** 2018-06-26

**Authors:** Haris Ahmad Khan, Sofiane Mihoubi, Benjamin Mathon, Jean-Baptiste Thomas, Jon Yngve Hardeberg

**Affiliations:** 1 The Norwegian Colour and Visual Computing Laboratory, NTNU–Norwegian University of Science and Technology, 2815 Gjøvik, Norway; jean.b.thomas@ntnu.no (J.-B.T.); jon.hardeberg@ntnu.no (J.Y.H.); 2 Le2i, FRE CNRS 2005, Université Bourgogne Franche-Comté, 21000 Dijon, France; 3 Univ. Lille, CNRS, Centrale Lille, UMR 9189—CRIStAL, Centre de Recherche en Informatique Signal et Automatique de Lille, F-59000 Lille, France; sofiane.mihoubi@ed.univ-lille1.fr (S.M.); benjamin.mathon@univ-lille.fr (B.M.)

**Keywords:** hyperspectral image, spectral analysis, effective dimension, spectral LBP, texture, dataset, reflectance

## Abstract

We present a dataset of close range hyperspectral images of materials that span the visible and near infrared spectrums: HyTexiLa (Hyperspectral Texture images acquired in Laboratory). The data is intended to provide high spectral and spatial resolution reflectance images of 112 materials to study spatial and spectral textures. In this paper we discuss the calibration of the data and the method for addressing the distortions during image acquisition. We provide a spectral analysis based on non-negative matrix factorization to quantify the spectral complexity of the samples and extend local binary pattern operators to the hyperspectral texture analysis. The results demonstrate that although the spectral complexity of each of the textures is generally low, increasing the number of bands permits better texture classification, with the opponent band local binary pattern feature giving the best performance.

## 1. Introduction

When electromagnetic waves are incident on a material’s surface, some part of it is absorbed and some of it is reflected back. The reflected electromagnetic waves from a surface depends on its material composition, physical and chemical properties, surface roughness and the geometry of incident and reflected waves. The behavior of reflected waves from a surface in a particular wavelength region is known as the spectral signature of that material. To obtain the spectral signature from a surface, devices like spectrophotometers and hyperspectral cameras are used. The advantage of hyperspectral devices is the acquisition of high resolution spectral and spatial information of a material or scene, whereas the spectrophotometer is able to acquire spectral information from one point of the selected surface.

For the acquisition of hyperspectral images, push broom cameras have been extensively used [1]. In the push broom imaging technique, line scanning of the scene is performed and the spectrogram of a particular scan is recorded on a charged couple device sensor. Another method for the acquisition of hyperspectral images is through the use of a liquid crystal tunable filter (LCTF) [2] and MEMS Tunable Fabry-Pérot Filters [3] on top of a monochrome sensor. The use of such filters provides direct imaging ability, and line scan of the scene is no longer required. The drawback of using such filters is the low speed of spectral sampling and the lower number of channels compared to push broom cameras. Recently, low cost hyperspectral imaging devices have been introduced that use off-the-shelf components for the conversion of an imaging sensor into a hyperspectral camera [4].

The analysis of hyperspectral images is performed for image data exploration, classification and quantification tasks. There are various multivariate analysis techniques that are applied to hyperspectral images for data decomposition, pre-processing and performing regression or classification analyses. Some of the methods for hyperspectral data analysis include the principal component analysis [5], linear discriminant analysis [6], partial least squares discriminant analysis [7], K-nearest neighbor algorithm [8], support vector machine [9], least-squares support vector machine [10], artificial neural network [11], deep recurrent neural network [12] and deep convolutional neural networks [13]. Hyperspectral imaging is widely used in a number of applications, e.g., remote sensing [14], medical diagnosis [15], cultural heritage [16,17], face recognition [18], food quality control [19], color management and printing [20,21]. A number of applications have been developed through the multivariate analysis of hyperspectral images, including the investigation of dried blood spots [22], the measurement of oil and protein contents in peanut varieties [23], the classification of muscle foods [24], the detection of defects on peaches [25] and numerous other applications.

Hyperspectral images contain more spectral information compared to color images. The high dimensionality of hyperspectral image data is an open challenge, and a trade-off between high information content and practical handling is often required. To evaluate this compromise and assess its performance, high quality data with high spatial and high spectral resolutions are required. Creating such a dataset takes time and effort, but it is necessary to the research community.

Considering these needs, we present a hyperspectral image dataset of 112 textured objects falling into five different categories. Images are reflectance data that span the visible and near infrared (NIR) parts of the electromagnetic spectrum. The data is referred to as HyTexiLa (Hyperspectral Texture images acquired in Laboratory). The idea behind creating this dataset is to provide a platform for the benchmark analysis of various applications and processing. The areas where this data could be used are in the fields of image processing, computational imaging and computer vision, such as surface identification, spatio-spectral analysis of textured surfaces, image sensor simulation, color reproduction, image relighting and so on. The availability of a high spectral and spatial resolution dataset will provide easy access for the evaluation of different techniques and will allow the results to be compared. In this paper, we present that dataset and analyze its spatial and spectral properties. We define the image acquisition protocol, the distortions that occur during the acquisition of the objects, the method for the correction of such distortions and the effect of such corrections. Our focus in this paper is to present a hyperspectral dataset to the community and to provide an analysis on our dataset as a benchmark for further research. We provide the methodology for pre-processing the raw hyperspectral data before using it for the intended applications. This paper is organized as follows: First we analyze the existing hyperspectral datasets and then, in Section 3, we discuss the hyperspectral image acquisition protocol and the processing of spectral data for addressing the distortions that occur during the acquisition. In Section 4, a spectral analysis of the data is provided. Section 5 consists of the texture analysis of the object surfaces in our dataset, followed by the conclusions.

## 2. Comparison of HyTexiLa with Existing Hyperspectral Datasets

Several hyperspectral image datasets are publicly available. In this section, we provide an overview of the existing hyperspectral image datasets that consist of close range images, including natural scenes and indoor images of objects. In this section, we provide a brief review of existing hyperspectral image datasets and compare them with the properties of our proposed dataset. We do not include the remote sensing datasets in this section, because remote sensing does not assume the same imaging model. We also exclude reflectance datasets obtained from pointwise spectrometers because they will not contain any spatial relationship between spectra as well as those multispectral image datasets which contain less than 10 channels.

Table 1 gives an overview of the existing hyperspectral image datasets. The datasets in [26,27,28,29,30,31,32,33,34] consist of outdoor scenes which include vegetation and buildings. The wavelength range in [26,30,34,35,36,37,38] is from 400 nm to 700 nm, while the datasets [27,30,31] have spectral ranges from 400 nm to 720 nm. The spectral sampling in [26,27,29,30,31,32,33,35,36,39] is 10 nm. The dataset in [28,32,40,41] has a spectral range of 400 nm to 1000 nm. The datasets in [35,36] consist of images of objects taken indoors. There are some datasets which consist of a specific category of objects, like paintings [42], leaves from apple tree [43], textiles [39], honey [41] and wood samples [44]. Most of the available datasets are sampled or re-sampled at 10 nm intervals, while some of them have different sampling intervals, e.g., [28] has 1.25 nm sampling step, while datasets in [41,43] have spectral sampling intervals of 6 nm and 4.7 nm, respectively.

In this paper, we provide a new dataset of hyperspectral images that has been made publicly available. This dataset consists of samples from five different categories of materials. The spectral range of this dataset is from 405.37 nm to 995.83 nm. As identified in Section 2, there are only a few datasets that span both the visible and NIR regions. The information in NIR is valuable for material classification [45,46,47], and the identification of textile fibers [48] and minerals [49]. Although some of the hyperspectral datasets provide information in the NIR region, they are either for specific samples, as in [41,42,43], or consist of outdoor scenes with many objects, as in [28,32]. Each image in our dataset consists of one specific, rather flat, object. The spectral resolution of data is also an important aspect to be considered. In most of the hyperspectral image datasets mentioned in Table 1, the spectral sampling interval is 10 nm, which is considered enough to representat the spectra [50]. A spectral sampling of 5 nm was recommended for Munsell spectra in [50], but it is also worth noting that more closely sampled data may be required when spectra are less smooth. One of such cases is the presence of spiky illumination spectral power distributions, where the use of more accurate spectral intervals is required. The availability of high resolution spectral and spatial data will allow the development and testing of advanced resampling methods. There is an existing dataset for textile samples [39] but it contains spectral information in the visible region and the sampling has intervals of 5 nm between the bands. Some datasets consist of food items [41,45] but do not provide high spatial resolution. Similarly, the datasets containing images of urban and natural scenes do not provide high spatial resolution for each material. The CAVE dataset [35] contains detailed images of various materials that are captured from close range, but they are only in the visible region.

Our dataset was spectrally sampled at about 3.19 nm and can be used with simulations of most fluorescent tube and light emitting diode-based illumination sources. The advantage of our dataset when compared with already available datasets is the availability of high resolution spectral and spatial data. The spectral range in our dataset covers both the visible and NIR regions. Each image in the dataset contains a unique material, and it can be used as ground-truth spectral data for surface identification applications. Our dataset is available for public use in the form of reflectance data in which each image is cropped to a uniform size. The raw data consists of radiance images captured along with the white diffuser and gray reflectance patch. Since the raw data is of large size (about 442 GB), we do not provide it publicly but if someone is interested in processing the raw hyperspectral data, it can also be provided upon specific request to the authors.

## 3. Image Acquisition and Processing

In this section, we present and discuss the objects being imaged and the hyperspectral image acquisition setup. The issues faced during image acquisition and protocols to correct for those issues are also part of this section. We describe how we correct for distortions inherent to the system and how we compute reflectance images from the input radiance data.

### 3.1. Notations

We first list the notations and conventions used in this article. Functions are usually denoted in roman font, with sets in calligraphic font and scalar variables in italic font. All indexes start from 0, and [A] denotes the set of integers {0,1,⋯,A−1}. B is the set of wavelengths ${\left\{\lambda^{k}\right\}}_{k\in [K]}$ which ranges from 405.37 nm to 995.83 nm at 3.19 nm intervals. The hyperspectral images are denoted in bold font and spectral channels in italic font. D, I and R, respectively, denote the uncorrected (distorted) radiance image, the radiance image and the reflectance image. These images are made of *K* channels of M×N pixels associated with *K* spectral bands, centered at wavelengths $\lambda_{k}\in B$. $D^{k}$, $I^{k}$ and $R^{k}$ denote the *k*-th channel of the respective hyperspectral images. A pixel coordinate, *p*, of an image is denoted by a couple of bounded integer values: $p=\left(x,y\right)\in \left[M\right]\times \left[N\right]\subset Z_{+}^{2}$. $D_{p}$ (resp. $I_{p}$, $R_{p}$) and D(x,y) (resp. I(x,y), R(x,y)) denote the vector (spectral) value of D (resp I, R) at pixel p=(x,y). Similarly, $D_{p}^{k}$ (resp. $I_{p}^{k}$, $R_{p}^{k}$) and $D^{k}\left(x,y\right)$ (resp. $I^{k}\left(x,y\right)$, $R^{k}\left(x,y\right)$) denote the values of channel $D^{k}$ (resp $I^{k}$, $R^{k}$) at pixel p=(x,y). $S={\left\{S\left(\lambda^{k}\right)\right\}}_{\lambda^{k}\in B}$ is a vector of values of the spectral power distribution of the scene illumination, S, sampled at each $\lambda^{k}\in B$. Finally, ⌊·⌉ denotes the nearest integer function; ⌈·⌉ denotes the ceiling function; 〈·,·〉 denotes the inner product; and ||·|| denotes the Euclidean norm.

### 3.2. Objects in the Dataset

The different objects chosen for the dataset were divided into five classes: textile, wood, vegetation, food and stone. These samples are a set of textured object surfaces that exhibit different properties while remaining fairly flat. The imaged objects were reasonably flat so that the blurring effect due to distance variations from camera was minimal during the scanning process. After the completion of image acquisition, we analyzed each image thoroughly and discarded a few samples which were too blurry. We also excluded materials that exhibited high gloss in the considered viewing conditions to maintain a simple imaging model, as in Equation (1), which allows easy computation of reflectance values:

(1) $$\forall k\in \left[K\right],\;\forall \left(x,y\right)\in \left[M\right]\times \left[N\right],\;I^{k}\left(x,y\right)=R^{k}\left(x,y\right)\cdot S\left(\lambda^{k}\right).$$

Finally, for this dataset we provide 65 textile samples of various types and colors. They were selected among textile samples provided by the humanitarian association *Don De Soie* located in Templeuve in France. In the wood category, there are 18 different samples of wood that can be found in the area around Mjøsa lake in Norway. There are 15 images for the vegetation category, which consist of leaves, flowers and one mushroom. The food category consists of 10 samples of tea, coffee and spices, and there are four samples in the stone category. Image labels include the material category, the acquisition sequence number and a specific name if the material has been precisely identified. For the textile, wood, and vegetation samples, the labels also include the color of the material (suffix _*colorname* if there are at least two samples of the same type but with different colors) and/or the acquisition side (suffix _*back* if the back of the material has been acquired together with the front). We provide the sRGB rendering of each image along with the dataset for natural visualization of the imaged object. The sRGB values were computed from colorimetric computation on the spectral data (see Section 3.7).

### 3.3. Acquisition Setup

**Figure 1:**
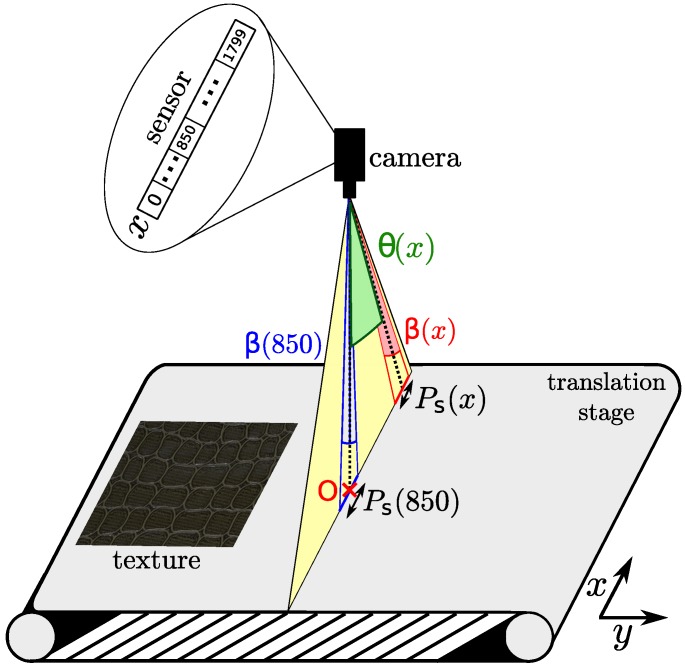
HySpex VNIR-1800 line scan acquisition process and cross-track distortion illustration on the *x*-axis. The camera acquires hyperspectral lines of pixels (*x*-axis). The hyperspectral image is obtained by translation of the object onto the *y*-axis.

We used a HySpex VNIR-1800 hyperspectral camera [51], manufactured by Norsk Elektro Optikk AS. This is a line-scan camera, consisting of a focusing mirror that allows only a narrow line of light in the scene to pass. This line is projected on a collimating mirror and then passed through a transmission grating. This grating separates the incoming light into different wavelengths, and each wavelength is focused onto a detector array (sensor). The camera is coupled with a close-up lens with a working distance of 30 cm. The output from that array consists of a slice of hyperspectral image, with spatial information in one direction and spectral information in the other. A combination of these line scans provide a full resolution hyperspectral image of the object or scene. The process of line scan is illustrated in Figure 1.

The output of the HySpex VNIR-1800 camera consists of a radiance image, D, made of K=186 channels associated with the spectral bands, centered at wavelengths $\lambda_{k}\in B$. The width of the acquired image is M=1800 which corresponds to the number of photodetectors along the spatial dimension of the detector array of the camera (*x*-axis), while the height, *N*, is the number of pixels in the scanning direction (*y*-axis) and is physically bounded by the size of the scene and the translation stage. During image acquisition, we observed spatial distortions in the output radiance images, which are addressed in the next section.

### 3.4. Corrections of Spatial Distortions

We observed a cross-track distortion on the *x*-axis and a shear distortion on the *y*-axis of the output images. These distortions were observed during the acquisition of test targets (grid images containing perfect squares, Macbeth ColorChecker, rulers). The correction of these distortions can be modeled by two functions, h:[M]×[N]→[M] and v:[M]×[N]→[N], so we set the value of the pixel given by (h(x,y),v(x,y)) of the distorted image to pixel (x,y) of the corrected image:

(2) ∀(x,y)∈[M]×[N],I(x,y)=D(h(x,y),v(x,y)).

The function h considers cross-track distortion and is called sensor correction since the distortion only depends on the physical properties of the sensor of the camera. This distortion is pointed out by the manufacturer (see Section 3.4.1). The function v considers the shear distortion. This distortion is due to a nonorthogonality between the sensor and the scan direction given by the translation stage. The function v is called affine correction, since the shear distortion can be modeled by an affine transformation (see Section 3.4.2).

#### 3.4.1. Cross-Track Distortion

The acquired images present a distortion across the scan direction called cross-track distortion. This distortion is due to the fact that each pixel is associated with a surface element whose size varies and is the same for each line of a channel and for each channel. Figure 1 shows the whole field of view of the camera for a given line of pixels (yellow triangle). Point O (red cross) is denoted as the intersection of the optical axis of the camera and the translation stage. The camera associates 850 pixels to the left size of O and 949 pixels to its right side, as can be seen on the sensor of the camera. However, the surface area observed on each side of O is the same. Consequently, the pixels on the left side will be associated with higher surface elements (compression) than the pixels on the right side (expansion). To overcome this distortion we use the sensor model function θ provided by the camera manufacturer that represents the field of view of each pixel in an acquired line of pixels. The whole field of view of the camera can be deduced as θ(1800)−θ(0)=0.29 rad. We use θ(x) to compute the width of surface element $P_{s}\left(x\right)$ (in mm) observed by each pixel as

(3) $$\forall x\in \left[M\right],\;P_{s}\left(x\right)=H\cdot \left(\tan\left(\theta (x)+\beta (x)/2\right)-\tan\left(\theta (x)-\beta (x)/2\right)\right),$$

where H=340 mm denotes the distance between the camera lens, and O, and β(x) represents the angular pixel size. The pixel size is undistorted at O (x=850) and β(850) is equal to $1.6\times 10^{-4}$ rad (data provided by the camera manufacturer). The angular pixels’ sizes β(x) can be computed using the following recurrence:

(4) $$\forall x\in \left[M\right],\;\beta \left(x\right)=\left\{\begin{array}{l} 1.6\times 10^{-4},\;\mathrm{if}\;x=850,\\ 2\theta (x+1)-2\theta (x)-\beta (x+1),\;\mathrm{if}\;x<850,\\ 2\theta (x)-2\theta (x-1)-\beta (x-1),\;\mathrm{if}\;x>850. \end{array}\right.$$

**Figure 2:**
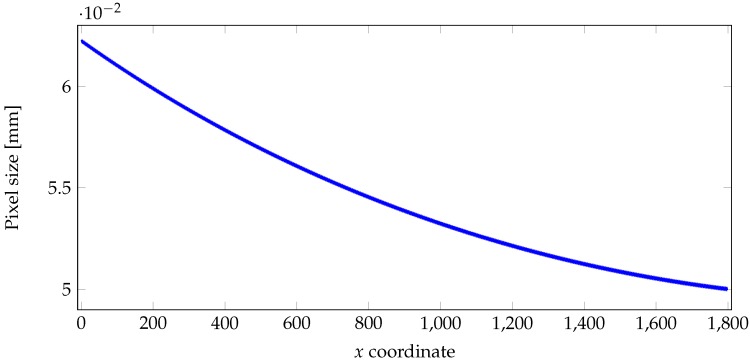
Size of surface element $P_{s}\left(x\right)$ (in mm) observed by each pixel of coordinate *x*. The values were computed using Equation (3), and the values of θ(x) were provided by the camera manufacturer.

Figure 2 shows the pixel size $P_{s}$ w.r.t. *x* coordinate. Note that $\sum_{x=0}^{1799}P_{s}\left(x\right)=98.6$ mm is the width observed by our camera.

To correct this distortion on acquired images, we measure the cumulative ratio $C_{r}\left(x\right)$ with respect to $P_{s}\left(850\right)$ as

(5) $$\forall x\in \left[M\right],\;C_{r}\left(x\right)=\sum_{i=0}^{x}\left(1-\frac{P_{s}\left(i\right)}{P_{s}\left(850\right)}\right).$$

Finally the sensor correction h is given by

(6) $$\forall \left(x,y\right)\in \left[M\right]\times \left[N\right],\;h\left(x,y\right)=x+\left\lfloor C_{r}\left(x\right)\right\rceil .$$

The pixel coordinate is rounded to the nearest integer to avoid artifacts due to the interpolation of pixel values.

#### 3.4.2. Shear Distortion

After the sensor correction, the image still exhibits a distortion on the *y*-axis due to the geometry of the set-up. Indeed, the direction to which the material moves and the line sensor are not exactly perpendicular. This implies that a rectangle in the scene becomes a parallelogram in the acquired image (shear distortion). This can be assumed to be an affine transform, leading to the correction

(7) ∀(x,y)∈[M]×[N],v(x,y)=⌊ax+by⌉,

and the transform only depends on two parameters: *a* and *b*. The pixel coordinate is again rounded here to the nearest integer to avoid interpolation artifacts. These parameters are experimentally estimated using the following method: we acquire 10 images of a grid with different rotations (the rotation angle ranges from 0 to π/4). Note that the grid is originally made of perfect squares: four equal sides and four equal angles (π/2 rad). Then, we apply the transform given by Equation (7) onto the grid images after sensor correction for different values for *a* and *b*. Finally, we measure the mean of the angles, γ, between the “horizontal” and “vertical” lines obtained after Otsu thresholding [52] and Hough transform [53] for all sensor-corrected grid images and three spectral channels, and retain the values of *a* and *b* that minimize |γ−π/2| (i.e., values of *a* and *b* that create right angles on grid images). We use the Otsu and Hough techniques here since they require few parameters and are adequate for the estimation of lines in these simple grid images which are just made of dark straight lines on a light background. The angular resolution for the Hough transform is set to $\arctan\left(2/1800\right)=1.2\times 10^{-3}$ rad and represents the smallest angle between two lines of pixels in an image acquired by our camera. Optimal values of *a* and *b* give

(8) ∀(x,y)∈[M]×[N],v(x,y)=⌊−0.021x+1.032y⌉.

Table 2 shows the mean and standard deviation of |γ−π/2| before and after applying the correction of Equation (8). It can be seen that, after the correction, the mean γ of the angles of the squares of the grid images is close to π/2 with a difference of $6\times 10^{-4}$ rad which is lower than the angular resolution ($1.2\times 10^{-3}$ rad).

**Figure 3:**
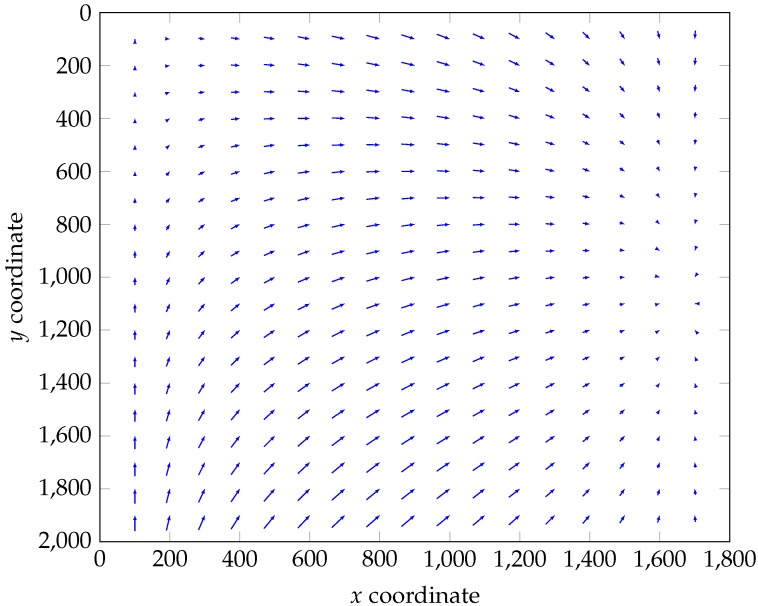
Vector field of both the *x*-axis and *y*-axis corrections. The origin of each vector is (h(x,y),v(x,y)), and its end is (x,y). For clarity, only vectors whose final coordinates (x,y) are multiple of 100 are shown here.

Figure 3 represents the vector field of the correction on an image of size 1800×2000 pixels. The origin of each vector is (h(x,y),v(x,y)), and its end is (x,y). Both corrections are translations along the *x*-axis (Equation (6)) or *y*-axis (Equation (8)), so they can be done jointly or separately. At the end, we obtain, for each acquisition, a corrected radiance image, I, made of K=186 channels.

### 3.5. Impact of the Corrections on Pixel Resolution

To quantify the impact of the sensor and affine correction on the pixel resolution, we acquire an image of a ruler with equally spaced markings of 1 mm along the *x*-axis. Then, we measure the pixel resolution in pixels·mm^−1^ thanks to the spaces between the markings of the single-channel image resulting from an average on 19 spectral channels without corrections—after sensor correction and after sensor and affine correction. Table 3 shows the means and standard deviations of these resolutions.

It can be seen that the pixel resolutions are similar, on average, before and after applying sensor correction (without corrections, pixel widths are higher to the left of intersection O between the translation stage and the optical axis than to the right, see Section 3.4.1), but the standard deviation is reduced by 2 along the *x*-axis which means that the sensor correction correctly decreases the cross-track distortion. Moreover, we notice that the pixel resolution, on average, is close to the theoretical one given by $1/P_{s}\left(850\right)=18.38$ pixels·mm^−1^ (see Equation (3)) and the affine correction does not impact the pixel resolution along the *x*-axis.

**Figure 4:**
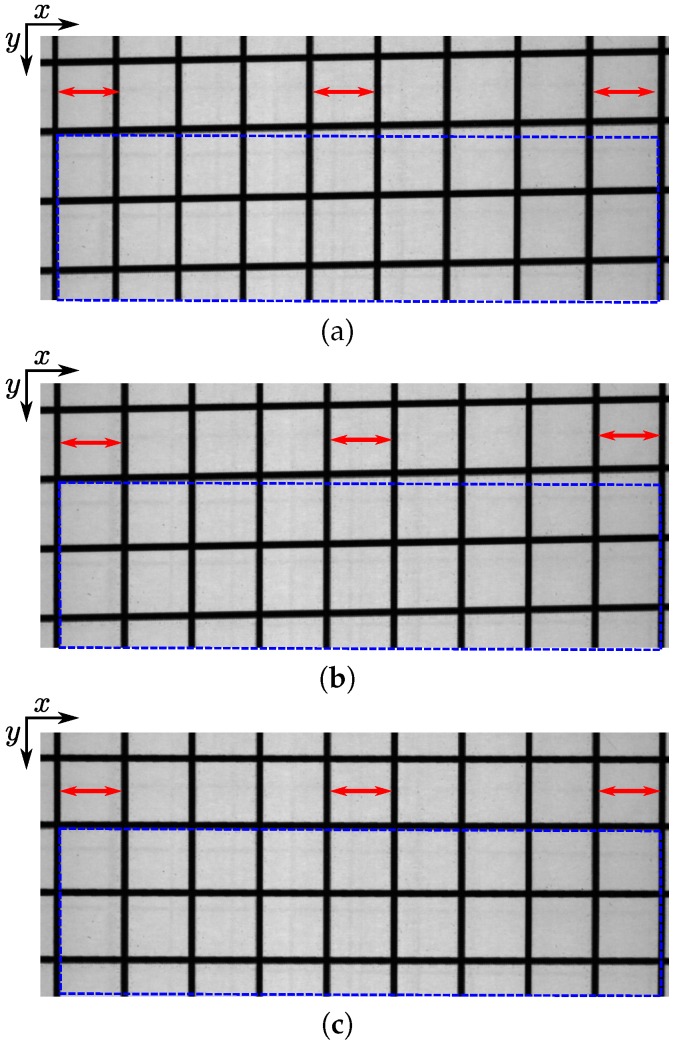
Images of the 49th channel of the acquisition of the grid made of perfect squares without corrections (**a**), after sensor correction (**b**) and after sensor and affine correction (**c**). Without corrections, the pixel resolution increases w.r.t. the *x* coordinate (sides of squares are higher to the right of the image than to the left). After corrections, the pixel resolution is constant w.r.t. the *x* coordinate (sides of squares remain constant). Moreover, the shear distortion is reduced after sensor and affine correction since the squares have right angles. The red arrows and blue rectangles have been added as overlays to obtain a better view of the distances and angles. Note that they are identical (same lengths) from one image to another.

To illustrate the impact of the corrections, images of the 49th channel (centered at 561.76 nm) of the acquisition of the grid made of perfect squares without corrections, after sensor correction and after sensor and affine correction are shown in Figure 4. It can be seen that without corrections, the pixel resolution increases w.r.t. the *x* coordinate (sides of square are higher to the right of the image than to the left). After sensor correction or both corrections, the pixel resolution is constant w.r.t. the *x* coordinate (sides of squares remain constant) which means that the cross-track distortion has been reduced. Moreover, we observe on the image after both corrections that the shear distortion has been reduced (squares have right angles).

### 3.6. Reflectance Computation

In this section, we describe how we obtain the reflectance image R of a texture from the corrected radiance image I. To properly estimate the reflectance on each surface element of a texture, we first estimate the spectra, $S={\left\{S\left(\lambda^{k}\right)\right\}}_{\lambda^{k}\in B}$, of the illumination system provided with the HySpex VNIR-1800. This illumination system (3900e DC Regulated ER Lightsouce) is manufactured by Illumination Technologies, Inc. [54]. For the estimation of the spectral power distribution of this illumination, we use the SG-3051 SphereOptics Diffuse Reflectance Tile [55] which provides a reflexivity of 99% in the range of [400,1000] nm. We then acquire each texture simultaneously with the SG-3051 tile to produce uncorrected radiance images D; then we correct them using Equation (2) to obtain radiance images, I. We noticed that the SG-3051 tile has a rough texture that produces shaded areas in the acquired images. To produce a robust estimation of the illumination, we adopted the following strategy for each acquired texture: we select a sub-image, $P={\left\{P^{k}\right\}}_{k\in [K]}$, of 550×550 pixels centered in the SG-3051 tile in the radiance image, I; then we compute the summation image, *P*, of all channels: $P=\sum_{k\in [K]}P^{k}$ and retain the set, Q, of the pixel coordinates (x,y), whose values in *P* are greater than the 95th percentile. Finally, the estimation of the illumination is given by taking, for each channel ($P^{k}$), the median value of the pixels in Q (to avoid the impact of saturated values):

(9) $$\forall k\in \left[K\right],\;S\left(\lambda^{k}\right)=\mathrm{Median}\left({\left\{P^{k}\left(x,y\right)\right\}}_{(x,y)\in Q}\right).$$

Now, we can compute the reflectance image, R, by dividing each channel of the radiance image, I, by its corresponding value in S, inverting Equation (1):

(10) $$\forall k\in \left[K\right],\;\forall \left(x,y\right)\in \left[M\right]\times \left[N\right],\;R^{k}\left(x,y\right)=\frac{I^{k}\left(x,y\right)}{S(\lambda^{k})}.$$

**Figure 5:**
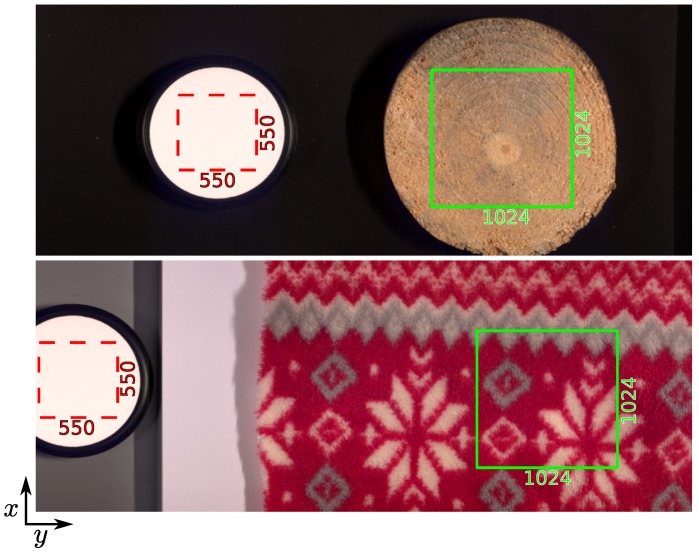
sRGB rendering of two corrected radiance images. The subimage (red dashed square) inside the SG-3051 reflectance tile is selected for illumination estimation and the texture region (green solid square) is retained as final reflectance image support.

Reflectance images are cropped to 1024×1024 pixels which corresponds to the minimum of the most relevant regions of interest (ROIs) of the 112 selected textures (ROIs that really contain texture elements). Figure 5 shows a sRGB rendering of two radiance images. For each radiance image, I, the subimage, P, selected for illumination estimation is displayed as a red dashed square, and the texture region that we retain as the reflectance image support, R, is displayed as a green solid square. Note that pixel values that have undergone specular reflection of the light in the radiance image can have values that are higher than the SG-3051 tile and produce values over one in the reflectance image. We decided to keep them unchanged in the final dataset so that the original corrected radiance image can be retrieved by multiplication with the original illumination spectra (which is provided in the metadata of the reflectance image file, see Section 3.7).

**Figure 6:**
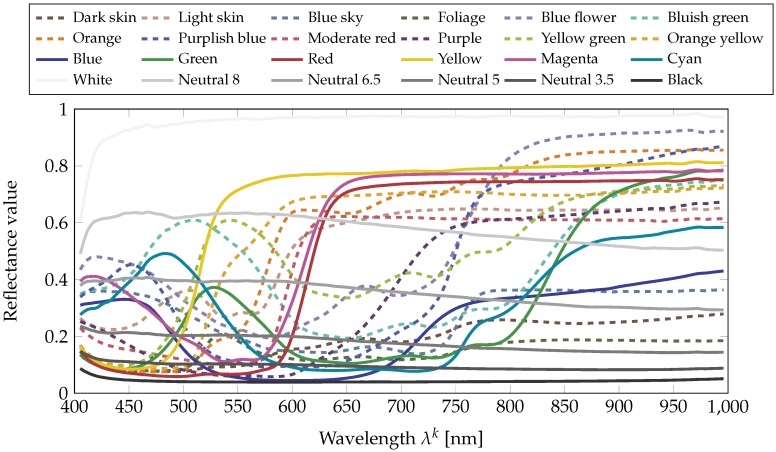
Spectral reflectances of the 24 patches of a Macbeth ColorChecker computed from the data acquired by the HySpex VNIR-1800 (on average on 200×200 pixels for each patch). The color of a curve is the sRGB color of the corresponding patch in the ColorChecker.

As a reference, we also compute the reflectance image of a Macbeth ColorChecker. Figure 6 shows the reflectances of the 24 color patches on average on 200×200 pixels for each patch. As for the reflectance measurements in [56], reflectance is mostly flat in the NIR.

### 3.7. Dataset Description

**Figure 7:**
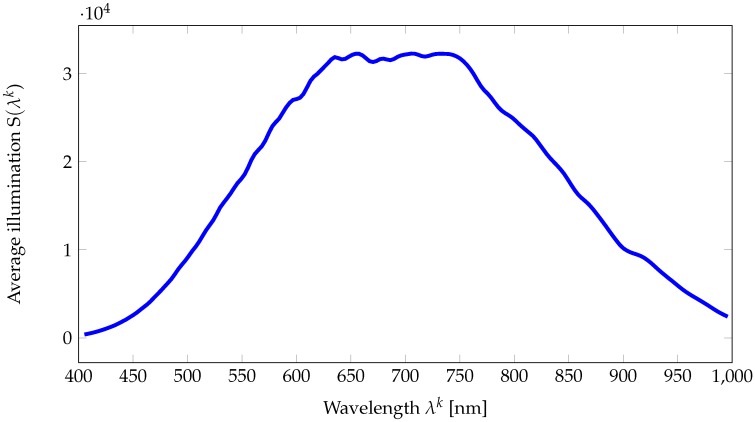
Average illumination computed over the 112 radiance images.

**Figure 8:**
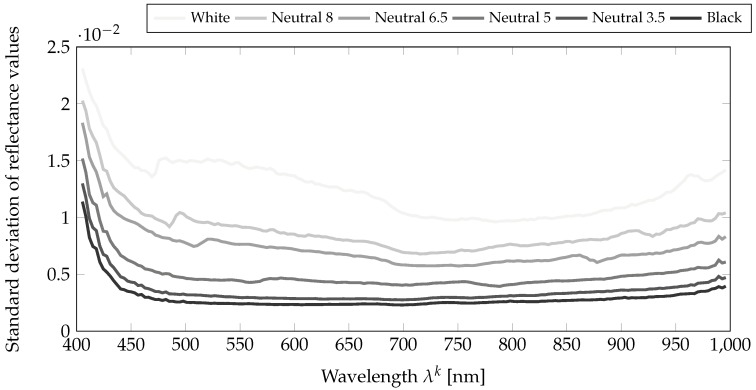
Standard deviations of spectral reflectances for 200×200 pixels of each gray patch of a Macbeth ColorChecker.

**Figure 9:**
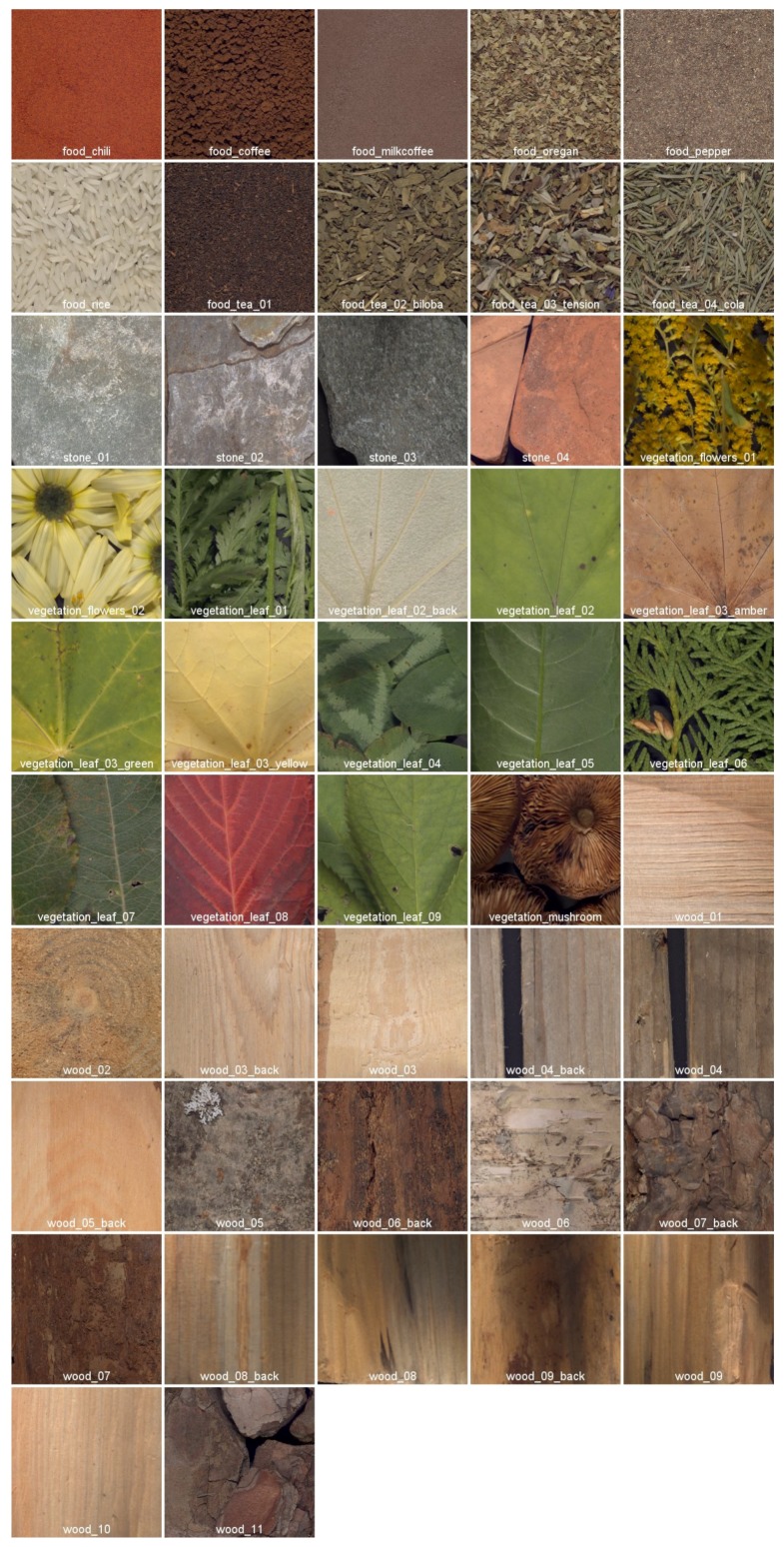
Reconstructed sRGB images from the food, stone, vegetation and wood categories of the dataset.

**Figure 10:**
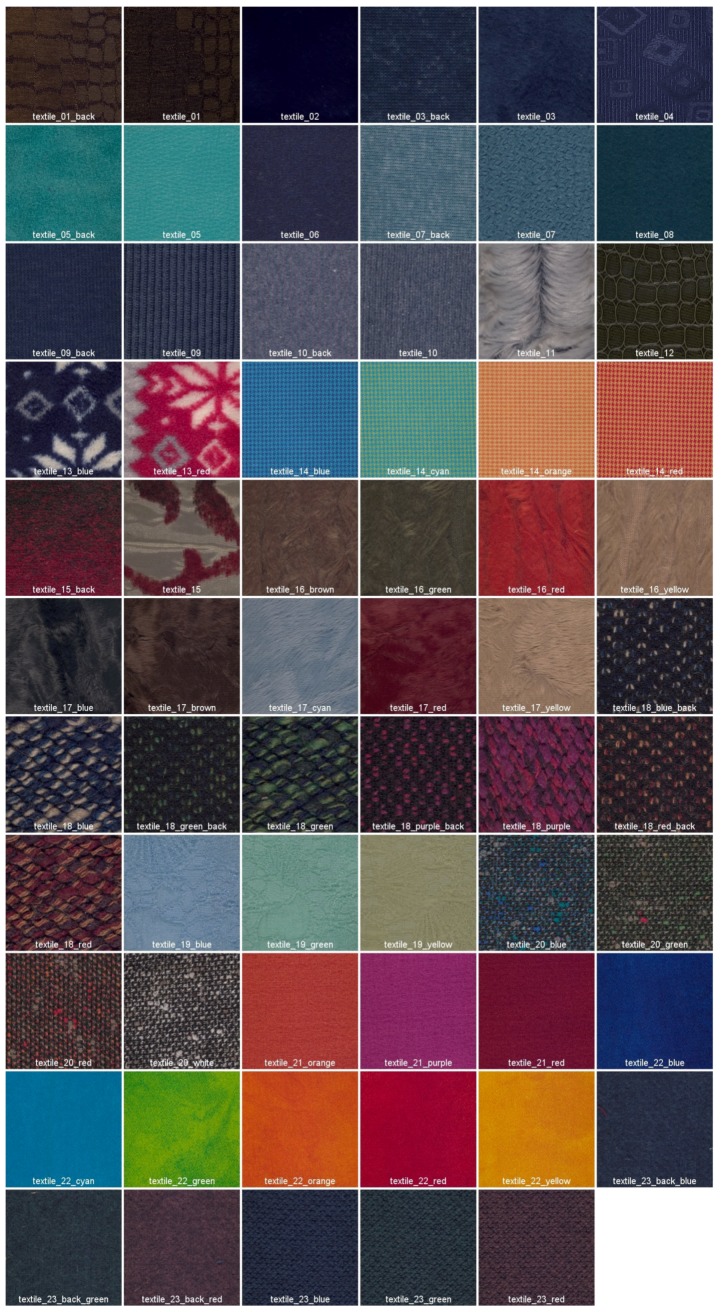
Reconstructed sRGB images from the textile category of the dataset.

The dataset is available on the websites of the team/laboratory of the authors (http://color.univ-lille.fr/datasets and https://www.ntnu.edu/web/colourlab/software). Each one of the 112 texture images is provided as both (i) a hyperspectral reflectance image in *ENVI* format and (ii) a sRGB reconstructed image in *PPM* format. (i) The *ENVI* format consists of two files: a binary file (extension *.raw*) of single-precision floating-point variables (32-bit) that stores the raw hyperspectral reflectance image in the *BSQ* (Band SeQuential) data organization (channel by channel) and a text file (extension *.hdr*) which contains the metadata of the image. The *.hdr* text file holds:

- A description of camera parameters (i.e., camera ID, integration time, aperture size, etc.). These metadata were written by the HySpex acquisition software and we decided to keep them unchanged.
- Required information to open the associated binary raw file (i.e., image size, number of bands, data type, etc.),
- Default bands (65, 45, and 18) whose band centers match with the primary Red, Green and Blue of sRGB standards. Generally these are used to generate false color RGB images using three channels.
- Wavelength array that contain values of $\lambda^{k}\in B$ in nanometers, for example, the center wavelength of channel $R^{k}$ is $\lambda^{k}$,
- Illumination array that contains the values of illumination ${\left\{S\left(\lambda^{k}\right)\right\}}_{\lambda^{k}\in B}$ provided by Equation (9). The illumination can be used to compute the corrected radiance channels, $I^{k}$, using Equation (10). The average value of this illumination over the 112 images is provided in Figure 7. It can be seen that illumination around 400 is weak, and consequently, respective channels in the reflectance images are likely to undergo noise [57]. We then measure the noise power by analyzing the standard deviation on 200×200 pixels on each gray patch of the reflectance image of the Macbeth ColorChecker on Figure 8. In addition to the illumination effect, the problem of having a good signal at low wavelengths is classically due to optics and low sensor sensitivity in this area, where we are at the limit of the optical model that is being used . Nevertheless we chose to provide these data and let the users decide if they want to use them in their simulations.
  Moreover, we provide ImageJ plugins and Python code for opening and processing the data. Matlab users can call the following instruction to open one hyperspectral reflectance image:
  R = multibandread(’<image_name>.raw’, [1024,1024,186],’float32’,0,’bsq’,’ieee-be’); These codes are available as Supplementary Materials.

(ii) The reflectance images are converted from the 186-channel domain to the sRGB color space. This transformation is performed using the CIE XYZ 2° standard observer functions. Then, the reference white D65 is used to convert values from XYZ to the sRGB color space. The resulting sRGB images are provided in *PPM* format. Figure 9 and Figure 10 show the 112 sRGB samples of textures with filenames.

## 4. Spectral Dimension Analysis

In hyperspectral images, the adjacent spectral bands are highly correlated [58]. Even though there are many good arguments for processing hyperspectral images at full spectral resolution [59], dimensionality reduction is often beneficial. It is performed by approximating the original hyperspectral data to a varying degree of precision with a smaller number of dimensions. The dimensionality or complexity of acquired reflectance spectra define the dimension of the vector space that is required to describe a spectrum.

There are various methods used for the computation of the effective dimension of spectral data, which can mainly be divided into supervised and unsupervised techniques. Supervised methods aim to preserve the a priori information, while unsupervised methods do not assume any prior information about the input data. Among the unsupervised methods, principal component analysis (PCA) [60,61] and independent component analysis (ICA) [62] are the most popular. PCA aims to maximize the variance of the input data by projecting it into the orthogonal subspace, while ICA looks for the source of changes in the input data, and these sources are assumed to be statistically independent from each other. Among the supervised methods, Fisher’s linear discriminant analysis (LDA) [63] and discriminative locality alignment (DLA) [64] are the prominent techniques, and many variants of these techniques have been proposed in the literature. A review of linear and non-linear dimensionality reduction methods can be found in [65].

Despite several previous studies being performed, there is still no agreement on the number of effective dimensions to represent the spectral data. There are different conclusions by various authors for the number of effective dimensions that can represent the spectral information. As an example, for the Munsell reflectance dataset, different authors have provided different dimensions that effectively represent it, ranging from 3 to 18 dimensional representations [66]. The required dimensionality of data mainly depends on the statistical properties of the spectra being considered and the information that one wants to preserve or emphasize. A review of the work done for finding the dimension of spectral data is provided in [66].

### 4.1. Spectral Analysis of the Proposed Dataset

In this section we investigate the effective spectral dimension of the acquired hyperspectral images. We do not assume any a priori knowledge about the data and provide a benchmark analysis in the spectral domain. Having these goals in mind, we used the principal component analysis (PCA) technique, which is mainly used to reduce the dimensionality of data consisting of a large number of inter-related variables, while retaining the variance in data [61]. PCA has the ability to de-correlate the spectral data and provide variance information through simple computational methods. This ability makes it one of the most popular techniques for the computation of the effective spectral dimension in hyperspectral imaging [67].

For the computation of effective spectral dimensions, each image is processed individually. First, a covariance matrix is computed, and then eigen decomposition is applied on this matrix to obtain the eigenvalues and eigenvectors. The eigenvectors are arranged relative to their eigenvalues in descending order. These eigenvectors consist of the characteristic reflectances of the input hyperspectral image. For each eigenvalue, corresponding eigenvectors exist, and a strong concentration of variance is present in the first few eigenvectors. If reflectance spectra are linearly independent, then the eigenvalues will all be significantly above zero. This shows that each wavelength contains independent information and is not redundant. However, in the case of hyperspectral imaging, typically, only a few eigenvalues are significantly higher, while the remaining values are close to zero, which indicates that their corresponding eigenvectors contain less variance and are mostly redundant. For an effective representation of the spectral data, we select only the first few eigenvalues, and the projection of spectra on the corresponding eigenvectors is able to provide adequate representation of the acquired data. This means that if we select the principal components containing 95% variance, the rest of principal components can be discarded with a loss of 5% of information. The decision to keep a certain percentage of the variance depends on the application for which a reduced set of representative data is required. For this analysis, we computed the effective dimension for each hyperspectral image which retained 99% and 95% of the total variance [66].

**Figure 11:**
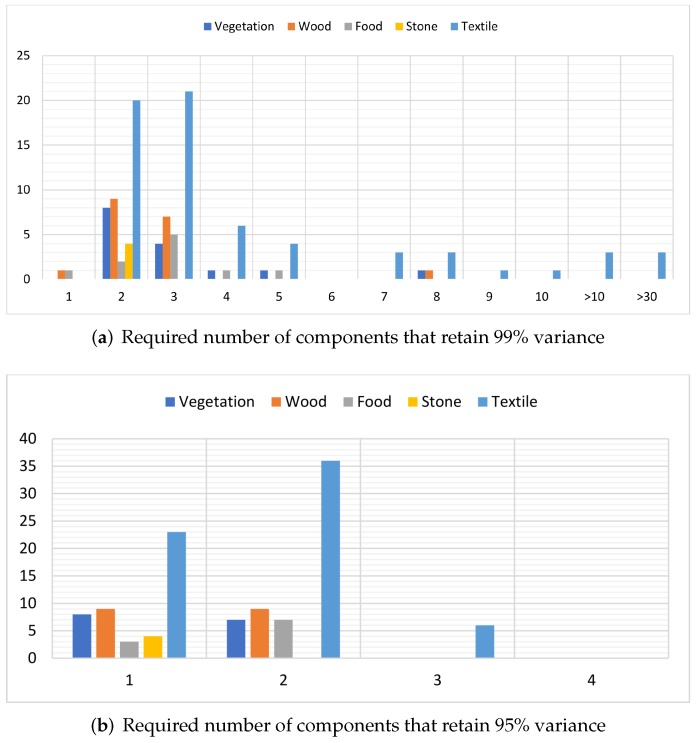
Results of the principle component analysis (PCA) on each image from the five categories of the hyperspectral dataset. The *x*-axis represents the effective dimension, while the *y*-axis shows the number of images with that effective dimension. Most of the spectral data in our dataset can be represented by 1, 2 or 3 dimensions while retaining 95% of the variance.

**Figure 12:**
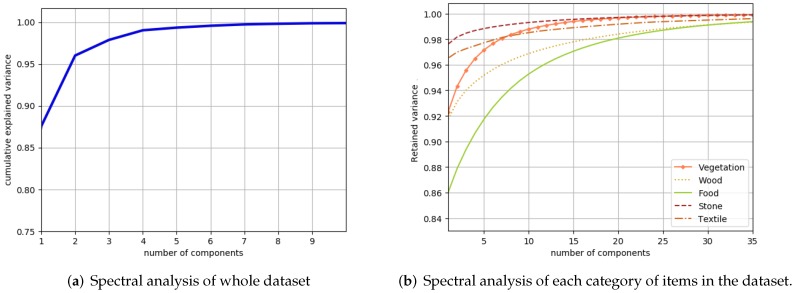
Number of components (x-axis) required to retain the variance percentage (y-axis). (**a**) shows the results for the whole dataset, while (**b**) shows the results for each of the five categories in the dataset. “Food” category images require more dimensions for their representation due to the variety of objects in that category, while those in the “stone” category require the least number of dimensions since the surfaces exhibit similar spectral properties.

Figure 11a shows the number of components required to retain 99% of variance, and Figure 12 gives information about the effective spectral dimensions of the whole dataset and also about each of the five categories of materials. It can be observed that most of the hyperspectral images show an effective dimension of two or three. All of the “stone” category images have an effective dimension of two, while most of the “food” category show an effective dimension of three. Most of the “textile” category images have an effective dimension between two and five, while some of the images have an effective spectral dimension beyond seven. Among them, three images show effective dimensions of 33, 47 and 58. We further examined the image that showed the highest number of dimensions (*textile_05* image) and found that 82.55% of the variance is retained by the first PC while 12.02% variance is retained by the second component. The rest of components add very little to the information, for example, the third PC contains 0.7% of the variance in this image. If the desired value of retained variance is reduced to 95%, then most of the images in our dataset can be defined with one or two principal components, while six images from the “textile” require a third component. Figure 12b represents the number of images that require a certain number of dimensions to retain 95% of the variance.

From these figures, it can be observed that the number of effective dimensions is dependent on the nature of the materials being imaged and may be used as a feature to estimate the category of imaged object. The reason why some of the images require a higher dimension for the description of their spectral properties is due to the presence of anomalies, for example, a tiny part with specular reflection which causes the effective dimension to rise. Figure 11 shows that by reducing the threshold for retained variance, the number of dimensions required to represent the spectral data is reduced.

### 4.2. Interpretation of the Effective Dimension

The effective dimension analysis of our dataset on the basis of PCA, as described in Section 4.1, provided a number indicating the number of dimensions required to retain a certain percentage of variance. However, it did not provide a physical meaning to the retained spectra since the principal components contain both positive and negative values. In order to obtain the information about the retained spectra, we used the non-negative matrix factorization (NMF) [68]-based decomposition on the hyperspectral images in our dataset. NMF is used in remote sensing hyperspectral images for spectral unmixing of the acquired data [69,70]. The advantage of NMF over PCA is that it extracts sparse and meaningful features from the given data, and the non-negativity constraint ensures the output is interpretable in terms of its physical meaning.

NMF is able to compute the spectral signature of end-members in a given hyperspectral image and the presence of each end-member in each pixel in the image in the form of abundance maps. The assumption behind this computation is the *linear mixing model* which states that the spectral signature of each pixel is a linear combination of the spectral signature of end members at a particular spatial location.

**Figure 13:**
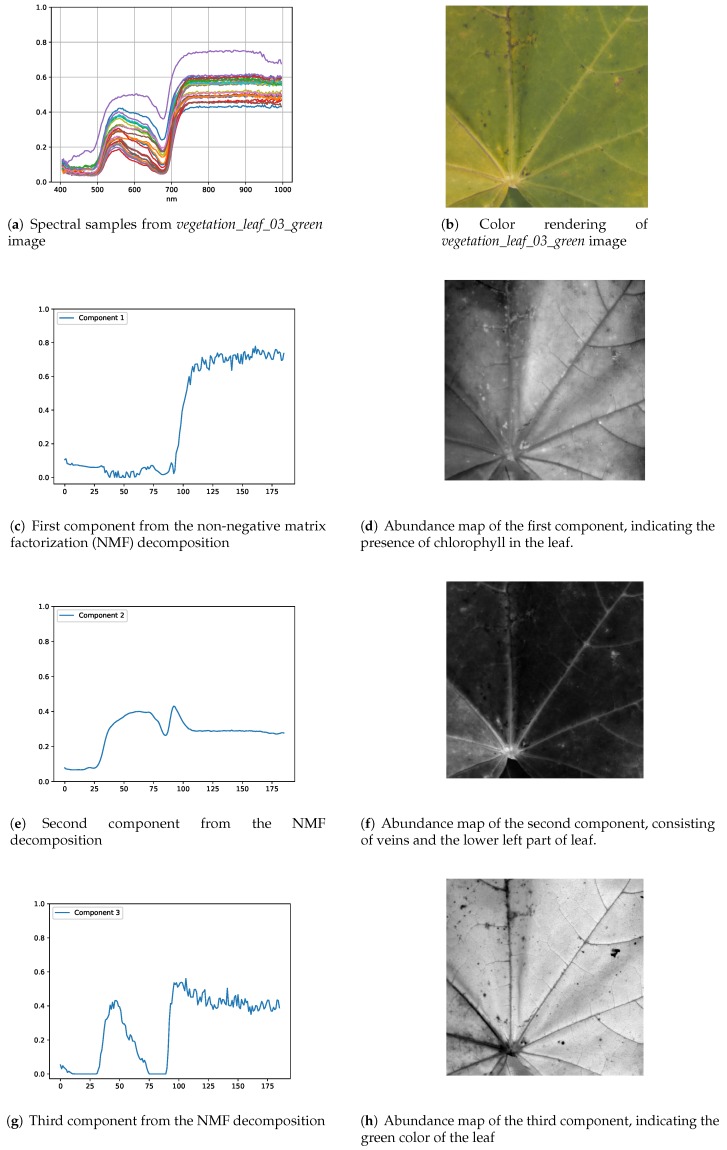
NMF-based decomposition of the *vegetation_03_green* image in three components. Each component exhibits the spectral composition of leaf, showing the spectra of chlorophyll, veins and the color of the leaf.

**Figure 14:**
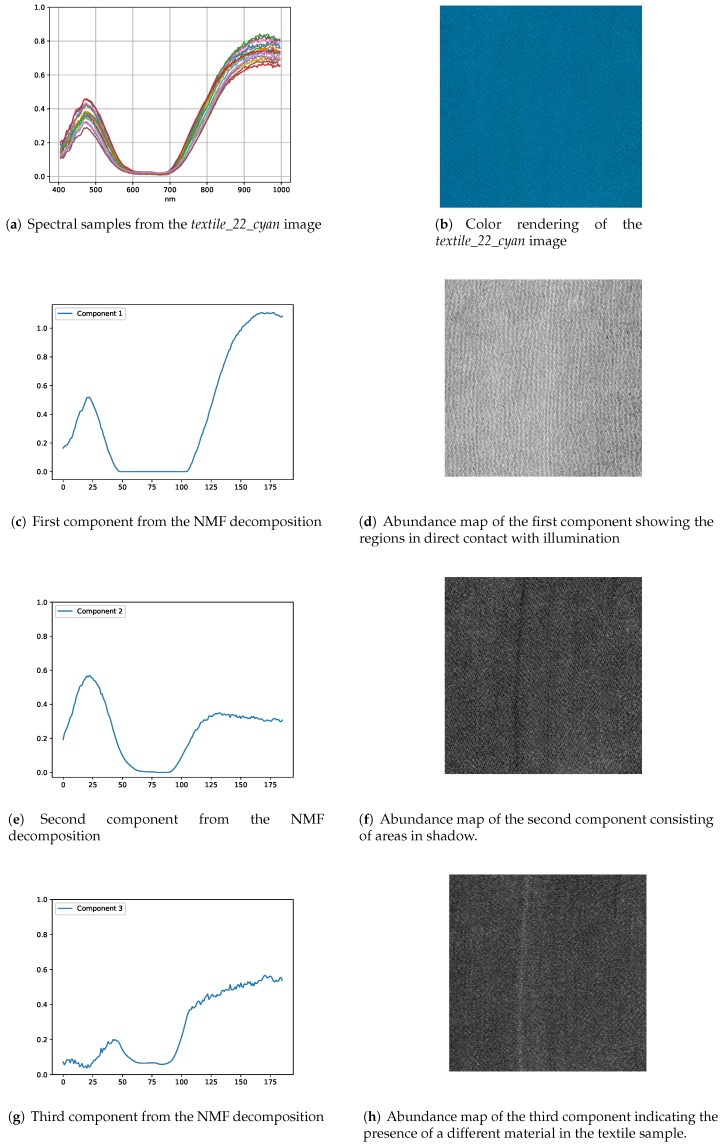
NMF-based decomposition of *textile_22_cyan* image in three components. The extracted components consist of areas under direct illumination, shadow areas and the presence of different materials in the textile sample.

In NMF-based decomposition, the number of components to be retained is provided by the user, which is mostly based on the prior knowledge of the end-members. For our dataset, we used PCA to compute the required number of components. For the illustration of NMF based decomposition results, we provide two examples in Figure 13 and Figure 14, containing samples of a leaf and textile, respectively. Figure 13b is the color rendering of the *vegetation_leaf_03_green* image from our dataset, and Figure 13c shows spectra from randomly selected pixels in this hyperspectral image. We decomposed the image into three components and the spectral signatures of computed components are shown in Figure 13c,e,g. The corresponding abundance maps in Figure 13d,f,h, show the presence of extracted components in the image. The first component in Figure 13c is significantly reflective in the near-infrared region, while showing a lower response in the visible region. This spectra suggests that this component is providing information about the chlorophyll in the leaf, and the abundance map in Figure 13d shows the areas where the presence of chlorophyll is prominent. The second component is formed by the veins and lower left part of the leaf, as can be seen in Figure 13f. The green color of leaf corresponds the the third component, as seen in Figure 13g, and the abundance map in Figure 13h provides information about the distribution of this component in the leaf.

Figure 14 shows the decomposition results of the *textile_22_cyan* image. The first and second components contain information about the reflectance from the textile surface which causes its particular color appearance. It is interesting to note that although the spectral signatures of first two components are almost the same in terms of shape in the visible region, they have different intensities in the near-infrared region. Upon closer inspection of the abundance maps in Figure 14d,f, it can be observed that the first component consists of directly illuminated regions while the second component is composed of regions in shadows. There is a third component in this sample which corresponds to a line in the abundance map (Figure 14h) and is probably caused by some component in the textile which is not easily visible in a color image.

Although we have provided only two examples from the NMF-based decomposition of the spectral data, the results indicate that such an analysis can provide interesting information about the spectral composition of the materials present in an image. For example, the amount of chlorophyll in vegetation, information about pigments and dyes in a textile, and the quality of food materials on the basis of their spectral analysis. We used PCA to get information about the spectral dimension before decomposing it through NMF. From the comparison of spectral behavior and the effective dimension, it can be seen that effective spectral dimension of most of the objects in our dataset is within two to eight. However, this dimension should not be directly related to the required number of spectral channels needed for acquisition. Although there are some studies which have aimed to find the number of filters that can best be used to acquire the maximum spectral information [71], there is no agreement on the optimum number of channels since it is dependent on the nature of surface spectral properties. Nevertheless, knowledge of the effective spectral dimension is useful for dimensionality reduction and obtaining the components of spectra by decomposing it. In the next section, we study the impact of dimensionality reduction on classification performances on the hyperspectral dataset using Local Binary Pattern (LBP)-based descriptors.

## 5. Texture Classification

A texture image is a characterization of the spatial and spectral properties of the physical structure of a material or an object. A texture analysis can be seen as a set of measures that quantify and provide information about the spatial arrangement of the spectral responses of an object. During texture classification, the goal is to assign an unknown sample texture image to a known texture class. For this purpose, discriminant texture features are extracted from test images and compared to those extracted from training images whose classes are known. To extract a feature from an image, a descriptor is needed. The Local Binary Pattern (LBP) method is one of the most robust feature descriptors, and characterizes the local level variation in a neighborhood of each pixel. Due to its discrimination power and computational efficiency, LBP has become a popular approach for various applications [72]. LBP-based texture classification was first performed on gray-level images, since the original operator only used the spatial information of texture [73]. Later, Palm [74] showed that classification based on a color analysis outperforms the single use of intensity spatial information. Thus, texture feature extraction is extended to the color domain by taking both spatial and color textural information into account. Here, we propose to extend the color LBP-based descriptors to any *K*-channel radiance image, I. Then, we propose to study the discriminative power of these descriptors on our dataset with respect to an increasing number of channels.

### 5.1. Texture Features Based on Local Binary Patterns

The LBP operator, [73], characterizes the level variation in the neighborhood, $N^{P,d}$, made of *P* pixels at a spatial distance, *d*, from each pixel, *p*, of a gray-level image. The outputs of the LBP operator over the multi-channel image are summarized as a concatenated histogram to form a texture feature. Here, we extend four color LBP operators to hyperspectral LBP operators so that the LBP operator is applied on a *K*-channel radiance image, I. This radiance image, I, is rendered using both a reflectance image, R, for which values above 1 (that correspond to specular surfaces) are clipped to 1 and quantized on an 8-bit, and the standard illuminant, E, lights the whole spectrum uniformly. Note that we only hold the reflectance information by using the illuminant, E, to allow independence from the illumination properties in our study. Moreover, data are quantized on 8-bits since channels of digital radiance and color images are often quantized on integer values.

We now detail each LBP operator and how their radiance value, $I_{p}^{k}$, of a channel, *k*, compares with those in the neighborhood, $N^{P,d}$, with respect to the value of *p*.

- ***Marginal LBP (LBP):*** The basic LBP operator is applied marginally to each channel $I^{k}$ at each pixel *p* as [75]
  (11) $${\mathrm{LBP}}^{k}\left[I\right]\left(p\right)=\sum_{q\in N^{P,d}}s\left(I_{q}^{k},I_{p}^{k}\right)\cdot 2^{\epsilon (q)},$$
  where ϵ(q)∈[P] is the index of each neighboring pixel, *q*, of *p* in $N^{P,d}$, and the sign function, *s*, is defined as s(α,β)=1 if α≥β, or 0 otherwise. The final texture feature results from the concatenation of the *K* $2^{P}$-bin un-normalized histograms of ${\left\{LBP^{k}\left[I\right]\right\}}_{k\in [K]}$, and its size is $K\cdot 2^{P}$.
- ***The Opponent Band LBP (OBLBP):*** Mäenpää et al. [76] improved the LBP operator by taking the inter-channel correlation into account. For this purpose, they considered the opponent band (OBLBP) operator of each pair of channels, $\left(I^{k},I^{l}\right)$, $\left(k,l\right)\in {\left[K\right]}^{2}$:
  (12) $${\mathrm{OBLBP}}^{\left(k,l\right)}\left[I\right]\left(p\right)=\sum_{q\in N^{P,d}}s\left(I_{q}^{l},I_{p}^{k}\right)\cdot 2^{\epsilon (q)}.$$
  The final texture feature results from the concatenation of the $K^{2}$ histograms of ${\left\{{\mathrm{OBLBP}}^{\left(k,l\right)}\left[I\right]\right\}}_{k,l\in [K]}$ and its size is $K^{2}\cdot 2^{P}$.
- ***Luminance-Local Color Contrast LBP (L-LCCLBP):*** This approach considers an image to have both spatial information of luminance and inter-channel information of different bands. The spatial information of the luminance results from the LBP operator being applied to the pseudo panchromatic image (PPI) $I^{PPI}$, which is computed as the average value over all channels at each pixel [77]:
  (13) $$I^{PPI}=\frac{1}{K}\sum_{k\in [K]}I^{k}.$$
  Regarding the inter-channel content, Cusano et al. [78] define the local color contrast (LCC) operator that depends on the angle, Θ, between the value of a pixel, *p*, and the average value, ${\bar{I}}_{p}=\frac{1}{P}\sum_{q\in N^{P,d}}I_{q}$, of its neighbors in the spectral domain:
  (14) $$\Theta \left[I\right]\left(p\right)=\arccos\left(\frac{\langle I_{p},{\bar{I}}_{p}\rangle }{\left|\right|I_{p}\left|\right|\cdot \left|\right|{\bar{I}}_{p}\left|\right|}\right).$$
  The LCC operator is then given by
  (15) $$\mathrm{LCC}\left[I\right]\left(p\right)=\left\{\begin{array}{cc} \left\lceil \frac{2\cdot 255}{\pi }\Theta \left[I\right]\left(p\right)\right\rceil & \mathrm{if}\;0\leq \Theta [I](p)\leq \pi /2,\\ 255 & \mathrm{otherwise}. \end{array}\right.$$
  The final texture feature is the concatenation of the histogram of $\mathrm{LBP}[I^{PPI}]$ and the histogram of LCC[I], and its size is $2\cdot 2^{P}$.
- ***Luminance-Opponent Band Angles LBP (L-OBALBP):*** As for L-LCCLBP, this approach first applies the LBP operator to the PPI $I^{PPI}$, and then Lee et al. [79] considers the angle between each pair of bands, $\left(k,l\right)\in {\left[K\right]}^{2},k\neq l$, as
  (16) $$\mathrm{OBALBP}{\left[I\right]}^{\left(k,l\right)}\left(p\right)=\sum_{q\in N^{P,d}}s\left(\arctan\left(\frac{I_{q}^{k}}{I_{q}^{l}+\eta }\right),\arctan\left(\frac{I_{p}^{k}}{I_{p}^{l}+\eta }\right)\right)\cdot 2^{\epsilon (q)},$$
  where η is a constant of small value. The final texture feature is the concatenation of the histogram of $\mathrm{LBP}[I^{PPI}]$ and the K(K−1) histograms of ${\left\{{\mathrm{OBALBP}}^{\left(k,l\right)}\left[I\right]\right\}}_{k\neq l\in [K]}$. Its size is $\left(1+K\left(K-1\right)\right)\cdot 2^{P}$.

### 5.2. Assessment on Our Proposed Dataset

#### 5.2.1. Covariance Analysis

Since 186 channels is too many to perform the proposed LBP descriptors, we propose to spectrally downsample the 112 radiance images. We selected, for each radiance image, I, *L* channels whose indexes uniformly ranged in [K]. The choice of a uniform distribution of channels was made to comply with the datasets and hyperspectral devices presented in Table 1 that provide spectral channels whose associated bands are uniformly distributed in their respective spectral range. The output is a spectrally sampled radiance image, $I_{L}={\left\{I^{k}\right\}}_{k\in C_{L}}$ where $C_{L}={\left\{\left(l+1\right)\left\lceil \frac{K-1}{L+1}\right\rceil \right\}}_{l\in [L]}$. Note that respective band centers, ${\left\{\lambda^{k}\right\}}_{k\in C_{L}}$, range uniformly in ]405.37,995.82[ nm.

**Figure 15:**
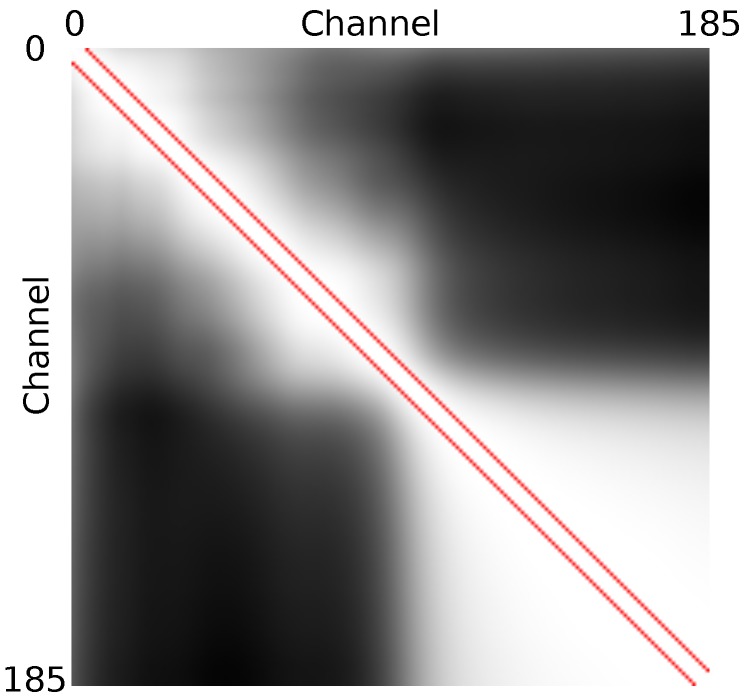
Normalized covariance between each couple of channels over the whole dataset. Values range between 31% (**black**) and 100% (**white**). The two red lines are separated by seven channels, and inside the red lines, the covariance is above 95%.

In order to determine the minimum number of channels, *L*, required to provide the best discrimination between channels, we studied the normalized covariance matrix between each couple of channels over the whole dataset computed in Section 4. Figure 15 shows that close channels in terms of band center wavelength are more correlated than distant ones and that infrared channels are highly correlated. The area inside the two red lines represents the covariance between channels with a distance of less than seven channels. In this area, the inter-channel covariance is above 95%. Stating this, we consider that a spacing of seven channels (22.33 nm) is enough, so in our study we considered a number of channels, *L*, that ranged from 1 to 27 channels. In colorimetry and in the colour imaging communities, it has been accepted that sampling spectra at 10 nm provides enough discrimination [80]. It is interesting to note that in our case, such large steps would provide a good description.

#### 5.2.2. Classification Scheme

A texture feature assessment was performed on our dataset by considering the 112 texture images as 112 different classes. Each 8-bit hyperspectral radiance texture image, $I_{L}$, of size 1024×1024×L was divided into 25 subimages of size 204×204×L (without overlap), among which 12 were randomly considered to be training images and the 13 others as test images. Supports of subimages according to this random sampling and corresponding classes are available as Supplementary Material as two text files (*train.txt* and *test.txt*). In a learning phase, LBP histogram features were extracted from each training image. Then, to assign a test image to one of the classes, the same features were extracted from each test image and compared to those of each training image. This comparison was performed using a similarity/dissimilarity measure between test and train features. Finally, each test image was assigned to the class of the training image with the best match by using a decision algorithm. The performances of a classification algorithm are determined by the rate of well classified test images, and depend on three main parts of classification, namely, the choice of discriminating textural features, the feature similarity measure and the decision algorithm. In order to determine the most discriminant texture features with respect to the number of channels, we propose retaining the 1-Nearest Neighbor decision algorithm coupled with the similarity measure based on intersection between histograms [81], since this classification scheme requires no additional parameter. Moreover, for simplicity, we propose using the LBP neighborhood, $N^{P,d}$, composed of P=8 pixels at a spatial distance of d=1.

#### 5.2.3. Classification Accuracy

**Figure 16:**
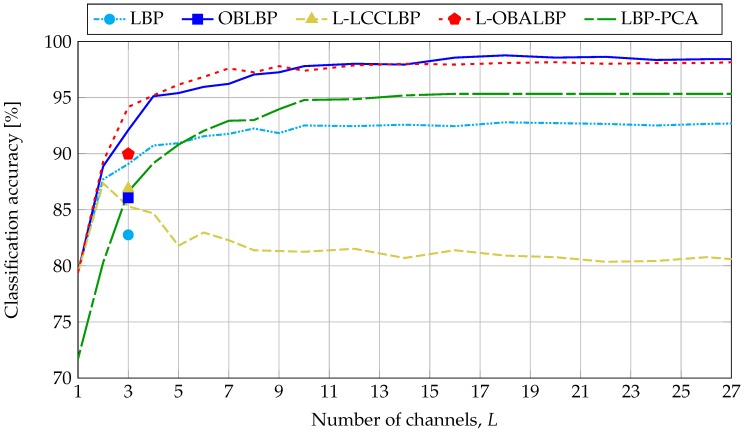
Classification accuracy reached by each Local Binary Pattern (LBP) descriptor with respect to the number of channels, *L*. LBP [73] (Equation (11)), Opponent Band LBP (OBLBP) [76] (Equation (12)), Luminance-Opponent Band Angle LBP (L-LCCLBP) [78] (Equation (15)), L-OBALBP [79] (Equation (16)). LBP-PCA refers to the marginal LBP operator applied to the PCA channels. Classification performances were stabilized after L=10 channels: about 92% accuracy for LBP, 98% accuracy for OBLBP and L-OBALBP, 81% accuracy for L-LCCLBP and 95% accuracy for LBP-PCA. The best performance was given by OBLBP with a 98.76% accuracy for L=18 channels. The markers refer to the classification accuracies of the descriptors applied on the L=3 channels of simulated RGB images from the three spectral sensitivities of the tri-CCD Basler L301kc color camera [82].

Figure 16 shows the classification performances reached by each texture feature presented in Section 5.1 with respect to the number of selected channels, *L* (see Section 5.2.1). The results show that, except for L-LCCLBP, increasing the number of channels improves the performance, and after 10 channels, the performance stabilizes. The marginal LBP operator, which takes only spatial correlation into account, reached about 92% accuracy after 10 channels. By taking spectral correlation into account, using information from opponent bands, the OBLBP and L-OBALBP operators reached about 98% accuracy after 10 channels. Regarding the L-LCCLBP operator, the performance was reduced, since instead of using spatial and spectral correlations for each channel, this operator uses only projected information on the panchromatic intensity. Overall, the results improved from 79.40% (LBP, OBLBP, L-LCCLBP and L-OBALBP with L=1) to 98.76% (OBLBP with L=18). In order to highlight the interest of hyperspectral classification against the color classification, we simulated color images using the three spectral sensitivities of the tri-CCD Basler L301kc color camera [82] and E illumination. The results show that, for three operators, the selection of three narrow spectral bands (two in the visible domain and one in the infrared domain) improves the performance compared to the selection of three wide bands in the visible domain.

We conclude that by increasing the number of channels, LBP descriptors provide more discriminative information about spatial and spectral correlations at the expense of feature size and computation time, and except for L-LCCLBP, hyperspectral imaging improves the discriminatory power of descriptors compared to color imaging.

In order to study the influence of channel distribution, we selected 10 sets of 10 random channels. All of these provided performances that were less than or close to performances obtained with a uniform distribution of 10 channels for the marginal LBP operator. We also considered the 10 channels with the highest variance among our dataset. They provided weak classification performances compared to uniform or random distributions, since all the related band centers are in the near infrared domain and consequently, do not take into account the textural information observed in the visible range.

**Figure 17:**
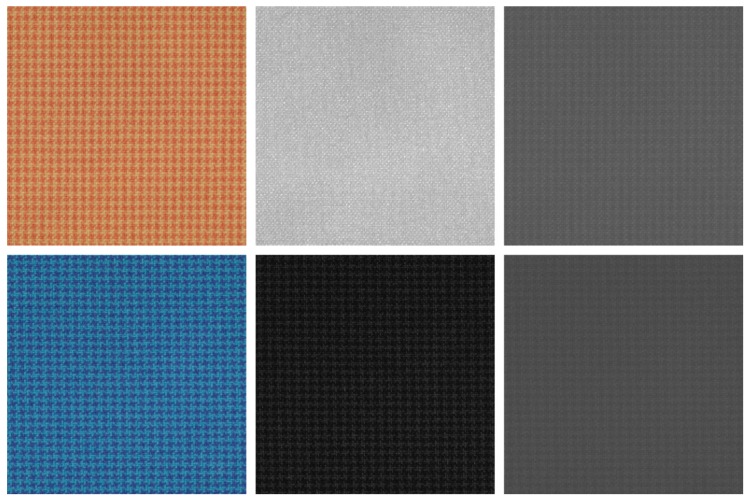
Texture image sample: *textile_14_orange* (top) and *textile_14_blue* (bottom). From the left to the right : sRGB rendering, 93th channel (centered at 699 nm) and 1st PCA channel. The images of the 93th channel of the two textures have different spatial properties and intensities so they can be easily discriminated, whereas the 1st PCA channels of both textures have similar spatial properties and intensities so they can barely be discriminated.

In order to study the discrimination of PCA for hyperspectral texture classification, we propose the application of the marginal LBP operator over the projections of the 186-channel hyperspectral images on the principal axis of the covariance matrix (denoted as PCA channels in the following). Note that the OBLBP, L-LCCLBP and L-OBALBP operators are not relevant for this study since they use spectral correlation which, by definition, does not exist between PCA channels. PCA channels are computed for each image of the dataset by first projecting the 186-channel reflectance image onto each principal axis, such that the first PCA channel is associated with the eigenvector with the highest eigenvalue and so on. For a fair comparison with the previous operators, PCA channels are then quantized on 8-bits using the same quantization function for all PCA channels of all 112 images. The LBP-PCA accuracy is shown Figure 16, where *L* is the number of sorted (according to decreasing eigenvalues) PCA channels that are considered for the LBP operator. By taking only the PCA channel with the highest variance (1st channel), classification performances are lower than by taking one real channel (93th centered at 699 nm). This can be explained by the specificity of our dataset. Since PCA is a projection of all channels into one channel, two similar textures with different spectral properties are barely discriminated using the PCA. For illustration purposes, Figure 17 shows the sRGB renderings, the 93th channels and the 1st PCA channels of two textures that are spatially similar but spectrally different (*textile_14_orange* and *textile_14_blue*). As can be seen, images of the 93th channel of the two textures have different spatial properties and intensities, so they can be easily discriminated, whereas the 1st PCA channels of both textures have similar spatial properties and intensities, so they can be hardly discriminated. By increasing the number of PCA bands, useful information from all channels is used so that it becomes more discriminative than taking each channel separately. In Figure 16, we can see that after five PCA channels, performances exceed the marginal LBP and after 10 PCA channels, the performances stabilize at about 95% accuracy. To conclude, using the PCA allows us to improve the performances by using only spatial correlation. However, in opposition to OBLBP and L-OBALBP which provide the best performances, it ignores the spectral correlation that allows the comparison of different channels, and thus, discriminates textures using their spectral properties.

## 6. Conclusions

In this paper, we introduced and described a hyperspectral reflectance dataset of close range textured object surfaces. The spectral range of this dataset is from 405 to 995 nm, thus providing spectral information in both visible and NIR regions. There are five classes of objects, and a total of 112 images are provided. We discussed the hyperspectral image acquisition protocol and the corrections that were applied to the data.

We performed a spectral analysis to quantify the spectral complexity of the samples and showed that a limited number of components permits a good description of their reflectance. Furthermore, we extended local binary pattern operators to a hyperspectral texture analysis. We observed that increasing the number of bands permits better texture classification. We also showed that the opponent band local binary pattern performed the best among the tested texture descriptors. Indeed, such descriptor uses an inter-channel correlation to discriminate textures using their spectral properties.

This dataset is available for scientific use and simulations, and it provides a benchmark to test various computer vision algorithms that are related to object classification and material identification. This dataset will also help in designing optimal spectral sensors, computational imaging systems and spectral reconstruction algorithms.

## Figures and Tables

**Table 1:** Summary of various characteristics of existing and our proposed hyperspectral image dataset (N/A means information not available from the dataset description).

Dataset	Nature of Images	Camera	No. of Images	Spatial Resolution	Spectral Range (nm)	No. of Channels
Bristol (1994) [26]	Outdoor scenes of vegetation	Pasecon integrating camera tube	29	256 × 256	400–700	31
Natural scenes (2002) [29]	Urban and rural scenes	Pulnix TM-1010 with VariSpec tunable birefringent filter	8	1024 × 1024	410–710	31
Natural scenes (2004) [30]	Reflectance image of natural scenes	Hamamatsu C4742-95-12ER with VariSpec liquid crystal tunable filter	8	1344 × 1024	400–720	33
Natural scenes (2015) [31]	Radiance image of natural scenes	Hamamatsu C4742-95-12ER with VariSpec liquid crystal tunable filter	30	1344 × 1024	400–720	33
Time-Lapse (2015) [27]	Images of 5 natural scenes taken at different times	Hamamatsu C4742-95-12ER with VariSpec liquid crystal tunable filter	33	1344 × 1024	400–720	33
ICVL (2016) [28]	Urban and rural scenes	Specim PS Kappa D×4	201	1392 × 1300	400–1000	519
Harvard (2011) [33]	Indoor and outdoor images	Nuance FX, CRI Inc.	50	1392 × 1040	420–720	31
UGR (2015) [32]	Outdoor scenes	Photon V-EOS	14	1392 × 1040	400–1000	61
CAVE (2008) [35]	Materials and objects	Apogee Alta U260 with VariSpec liquid crystal tunable filter	32	512 × 512	400–700	31
East Anglia (2004) [36]	Everyday objects placed in viewing booth	Applied Spectral Imaging Spectracube camera	22	Various resolutions	400–700	31
SIDQ (2015) [40]	Pseudo-flat objects	HySpex-VNIR-1600	9	500 × 500	400–1000	160
Brainard (1998) [37]	Indoor scenes	Kodak KA4200 CCD with Optical Thin Films filter	9	2000 × 2000	400–700	31
Nordic sawn timbers (2014) [44]	Wood samples	N/A	107	320 × 800	300–2500	440
Scien (2012) [45]	Various objects, scenes and faces	N/A	106	Various resolutions	Various range	Various channels
Paintings (2017) [42]	Paintings	IRIS II filter wheel camera	23	2560 × 2048	360–1150	23
Ancient manuscripts (2012) [38]	Printed documents	Hamamatsu C4742-95-12ER with VariSpec liquid crystal tunable filter	3	1344 × 1024	400–700	33
Apple tree leaves (2018) [43]	Near infrared images of healthy & infected leaves	HySpex SWIR-320m-e	N/A	Various resolutions	960–2490	256
SpecTex (2017) [39]	Textiles	ImSpector V8	60	640 × 640	400–780	39
Honey (2017) [41]	Honey samples	Surface Optic Corporation SOC710-VP	32	520 × 696	400–1000	126
Singapore (2014) [34]	Outdoor images of natural objects, man made objects, buildings	Specim PFD-CL-65-V10E	66	Various resolutions	400–700	31
HyTexiLa Our proposed dataset	Textured materials from 5 different categories	HySpex VNIR-1800	112	1024 × 1024	400–1000	186

**Table 2:** Mean and standard deviation of |γ−π/2| (in radians) on 10 grid images with different rotations before and after applying affine correction of Equation (8), where γ is the angle between a “horizontal” and a “vertical” line. It can be seen that the difference between γ and π/2 is strongly reduced (i.e., γ close to π/2) after the affine correction and is lower than the angular resolution ($1.2\times 10^{-3}$ rad).

	Value of |γ−π/2|
After sensor correction	0.0167 (0.0098)
After sensor and affine correction	0.0006 (0.0003)

**Table 3:** Means and standard deviations of pixel resolutions (in pixels·mm^−1^) measured between each marking or a ruler for a single-channel image resulting from an average of 19 spectral channels without corrections—after sensor correction and after sensor and affine corrections. It can be seen that the pixel resolutions are similar, on average, before and after applying the sensor correction, but the standard deviation is reduced by 2 along the *x*-axis which means that the sensor correction correctly decreases the cross-track distortion. Note that the affine correction does not impact the pixel resolution along the *x*-axis.

	Ruler along *x*-axis
Without corrections	18.30 (1.21)
After sensor correction	18.28 (0.60)
After sensor and affine correction	18.31 (0.61)

## Supplementary Materials

The following are available online at http://www.mdpi.com/1424-8220/18/7/2045/s1, (i) Hyperspectral images (can be downloaded from the websites of the team/laboratory of the authors: http://color.univ-lille.fr/datasets and https://www.ntnu.edu/web/colourlab/software). (ii) Two text files (*train.txt* and *test.txt*) which contain supports of subimages of the random sampling for supervised classification of Section 5.2.2 and corresponding classes (iii) ImageJ plugins (*MSTools-v18-05.zip*) and Python code (*falseColors.py*) for opening and processing the data.
